# Effect of Marriage and Spousal Criminality on Recidivism

**DOI:** 10.1111/jomf.12176

**Published:** 2015-03-04

**Authors:** Signe Hald Andersen, Lars Højsgaard Andersen, Peer Ebbesen Skov

**Affiliations:** ^1^ Rockwool Foundation Research Unit; ^2^ University of Copenhagen

**Keywords:** crime, marital quality, marriage, quantitative methodology, sociology

## Abstract

The authors analyzed whether the effect of marriage on recidivism varied by spousal criminality. For this purpose, they used propensity score matching and full population data from Statistics Denmark on all unmarried and previously convicted men from birth cohorts 1965–1985 (N = 102,839). The results showed that marriage reduced recidivism compared to nonmarriage only when the spouse had no criminal record. Similarly, marriage to a nonconvicted spouse reduced recidivism significantly more than marriage to a convicted spouse. These findings not only underline how important marriage is for social integration but also stress the heterogeneous nature of the protective effects of marriage.

Marriage is often considered an important lever for criminal desistance, especially among men (e.g., Sampson, Laub, & Wimer, 2006). But despite being guarded by a legally binding set of rules that apply to all marriages, marriage is not a uniform treatment administered similarly across couples. The dynamic of a specific marriage is likely to vary by the personality traits and characteristics of the two spouses as well as by their commitment to each other. When studies find that marriage reduces crime among men, this finding is thus likely to represent the average effect of the various types of marriages represented in the data, of which some have strong effects on desistance and some have small, no, or even opposite effects. A range of previous studies have shown, for example, that the effect of marriage on crime depends on the quality or strength of the marital ties (Farrall, Godfrey, & Cox, 2009; Laub, Nagin, & Sampson, 1998; Maume, Ousey, & Beaver, 2005; Rhule‐Louie & McMahon, 2007).

Scholars generally assume that part of the marriage effect works through the positive influence of the female spouse. Again, women may represent such positive influences to varying degrees and, given the predictions made by theories on assortative mating, convicted men may even wish to marry convicted women, who might be less likely to facilitate desistance. Cobbina, Huebner, and Berg (2010) presented evidence that only prosocial intimate partnerships reduce offending among females. Also, according to a recent Dutch study, marrying a spouse with a criminal history fails to decrease criminality among individuals who face conviction at some point during their lives (van Schellen, Apel, & Nieuwbeerta, 2012).

Our study contributes to this small literature on heterogeneous marriage effects by analyzing differences in recidivism between previously convicted men who marry convicted or nonconvicted women. For this purpose, we used methods suited for making causal inference and unique administrative data from Denmark. Our data provided information on all convicted men and contained a range of information on these men as well as on their spouses, including their own and their spouse's criminality, types of crime committed, important socioeconomic markers, and cohabitation history, all of which allowed for a rigorous test of how spousal criminality mediates the effect of marriage on recidivism.

Our results showed that marriage reduced recidivism compared to not marrying, but only among men who married nonconvicted spouses. There was no such marriage effect for men who married convicted spouses: Their recidivism did not differ from the recidivism observed among the nonmarried men. But, as could be expected, these men had significantly higher recidivism rates than men who married nonconvicted spouses.

## BACKGROUND

Marriage has a range of important implications. Studies have shown that marriage increases men's wages (e.g., Antonovics & Town, 2004) and improves their health (e.g., Dupre, Beck, & Meadows, 2009), just as there is a positive correlation between a person's marital status and his or her well‐being (e.g., Waite, 1995).

Equally important is that studies also have shown that marriage reduces antisocial and criminal behavior (e.g., Farrall, Godfrey, & Cox, 2009; Maume, Ousey, & Beaver, 2005; Sampson et al., 2006; Savolainen, 2009; van Schellen, Apel, & Nieuwbeerta, 2012), especially among young men (Theobald & Farrington, 2010). The literature presents us with two theoretical explanations of this effect: (a) Laub and Sampson's (2001) informal social control theory and (b) Warr's (1998) theory on peer association, which represents a later extension or alternative to the social control theory (see also Maume et al., 2005).

The *informal social control theory* originates in life‐course theory and builds on the Durkheimian claim that men are inclined toward antisocial behavior when nothing restricts them. Put directly, weak social bonds make way for antisocial behavior. Engaging in social institutions restricts such behavior because these institutions work as bonds to society and as mediators of social norms. For example, marriage implies social ties that redirect life trajectories otherwise bound for antisocial behavior, as it creates an interdependent system of obligations and restraints between the two spouses. In addition, social institutions are important sources of reward—which imposes significant costs for translating antisocial propensities into actions—and this is the reason why we are unlikely to just free ourselves from the marital bonds, despite their restraining nature. As a consequence, marriage causes desistence from crime. According to Laub and Sampson (Laub et al., 1998; Laub & Sampson, 2001), however, this effect depends on the strength or quality of the marital bonds, and if the bonds between the spouses are not strong the marriage will not increase social control.

The *peer association theory* also implies that marriage causes desistance from crime, but this theory poses a different explanation of the mechanism. Marriage reduces the amount of time a person (and, again, especially men) spends with peers, including delinquent peers. Also, because crime is usually a group activity or arises as a result of peer pressure (e.g., Hochstetler, Copes, & DeLisi, 2002), being away from delinquent peers reduces the risk of ending up in situations that enable or require criminal behavior (Giordano, Cernkovich, & Holland, 2003; Warr, 1998). Also, a female spouse may function as a role model that promotes a more prosocial way of life due to her daily routines and good examples, judgments, and positive feedback.

Whether the marriage effect arises from informal social control that binds the two spouses together or from a reduction of time spent with delinquent peers, both theories predict that marriage causes desistance from crime. Empirical studies support both explanations. First, Laub et al. (1998), Sampson et al. (2006), and Cernkovich and Giordano (2001) have demonstrated how the quality of the marriage matters for desistance. Second, Warr (1998), Simons, Steward, Gordon, Conger, and Elder (2002), and Giordano et al. (2003) have presented extensive evidence that changes in a person's relations to his or her peers explain away the marriage effect on crime. Third, Maume et al.'s (2005) findings supported both theories by showing direct effects of marriage even after controlling for—and getting significant estimates of—changes to the number of delinquent peers.

### *Marriage, Recidivism, and Spousal Criminality*

Neither the informal social control theory nor the peer association theory explicitly discusses how and to what extent the criminal history of the spouse affects recidivism, but we may still use both theories as frameworks for understanding the relationship. The informal social control theory implies that a marriage between two delinquent partners, who are unlikely to promote and adhere to law‐abiding norms, is an inefficient medium for transferring society's norms regarding delinquent behavior. Crime also represents less of a threat to this type of marriage, and criminal acts are then less likely to deprive the spouses of the rewards of marriage.

According to the peer association theory the marriage effect arises because marriage reduces the influence of delinquent peers and increases the influence from the spouse's good examples in everyday matters. But if the spouse is also delinquent, this may easily make up for the lack of such peers, and the spouse might become a bad influence herself. Also, a delinquent man, married to a delinquent spouse, may furthermore be less likely to rid himself of bad company because the spouse might more willingly accept these friends and even become integrated into the group (Simons et al., 2002). In sum, both theoretical perspectives predict a reduced or even absent marriage effect when a delinquent man marries a delinquent woman.

In addition to these theoretical predictions, a recent Dutch study used a sample of individuals convicted of criminal activities at some point in their lives (and not necessarily before their marriage) to show how the marriage effect was contingent on the criminal history of the spouse (van Schellen, Apel, & Nieuwbeerta, 2012). Although they focused on crime in general rather than recidivism, their finding is a strong signal that the effect of marriage on recidivism, too, depends on the criminal profile of the spouse. Furthermore, a recent study from Norway showed that offending rates changed around the time of marriage and that men who married a previously convicted spouse actually experienced a sharper decline before and after marriage in committed offenses than men who married a nonconvicted spouse (Skardhamar, Monsbakken, & Lyngstad, 2014). It is important to note that their results are descriptive, and their puzzling finding may reflect selection bias. Given the theoretical predictions as well as the findings of the Dutch study mentioned above, in this study we proposed and tested the following hypotheses: (a) The effect of marriage on recidivism is substantial (Hypothesis 1) and (b) the effect of marriage on recidivism is lower when the spouse has a criminal history (Hypothesis 2).

### *Selection Issues*

The key challenge when measuring the effect of marriage on recidivism is to account for selection into marriages. If one does not handle selection issues, one gets biased estimates of the marriage effect, and differences in the recidivism levels between married and unmarried men could arise from such preexisting differences in marriage potential and crime proneness rather than from their marital statuses. In our case such bias could arise from two selection issues: (a) what causes some to marry and others not and (b) what causes some to marry nonconvicted spouses and others to marry previously convicted spouses.

The first selection issue concerns reasons why people wish to marry, and why some people are more likely to marry. Here, theories state that marriage is attractive because it allows for the pooling of resources, it secures the stable company of others, and it provides a major setting for having children and safe and regular sex (G. Becker, 1991; Oppenheimer, 1988). In this case, marriage is desirable when the individual has and wishes to fulfill such needs. But, as is evident from the theories, both the likelihood of having these needs and the ability to fulfill them varies among people. First, people may have these needs to a smaller or larger extent, depending on age, norms, and life situations; young people may, for example, not yet feel a strong need for the pooling of resources, which means that the timing matters for people's desire to marry (Oppenheimer, 1988). Second, individual characteristics may increase or reduce the probability that one is able to fulfill these needs, that is, one's marriage potential (G. Becker, 1991). For example, having a criminal past could scare off potential spouses by signaling instability, lack of proper skills, and the lack of a sense of judgment. In sum, some people are less likely to marry both because of timing issues and because of their poorer marriage potential.

As a consequence, we observe systematic differences in the characteristics of married and unmarried men in cases where the married men have more favorable characteristics than unmarried men. Differences in the crime levels of these men might then arise from such preexisting differences in marriage potential and crime proneness as well as from their marital statuses. If we fail to account for the selection of men with favorable characteristics into marriage, we would then get an upwardly biased estimate of the marriage effect that would reflect both the actual causal effect of marriage on crime as well as these men's favorable characteristics, including their proneness for desistance. This means that we would overestimate the true effect.

The second selection issue concerns the mechanisms related to assortative mating. Theories on assortative mating and social homogamy imply that marriage to a convicted woman will signal that the man has less favorable characteristics than other married men. This is because convicted women are less attractive partners (Rhule‐Louie & McMahon, 2007; van Schellen, Poortman, & Nieuwbeerta, 2012). The postmarriage crime levels of such men then reflect both the marriage effect as well as their less favorable characteristics, characteristics that are likely to be correlated with a lower probability of desistance compared to other married men. In fact, one could speculate about whether the characteristics of these men are even less favorable than the characteristics of unmarried men. If we fail to account for selection for this group of men, the effect of marriage compared to nonmarriage would then look less positive than it actually is because of their lower initial propensity of desistance. Furthermore, if men who marry convicted women have less favorable characteristics than unmarried men, the selection issue may even cause the estimate to become negative. In both cases, the estimated marriage effect would reflect the causal effect of marriage as well as these men's reduced proneness for desistance, which means that we would underestimate the true effect.

This selection issue also implies that differences between the marriage effect of men married to nonconvicted women and the marriage effect of men married to convicted women (which we tested in our Hypothesis 2) would become artificially large. This happens because the favorable characteristics of the former group relative to the less favorable characteristics of the latter group will increase the distance between the two groups and boost the size of the estimate. As a result, we would overestimate the difference between the two groups, and this would cause us to wrongfully accept Hypothesis 2.

In the absence of a proper identification strategy, these various selection issues hinder causal inference, which would prevent us from estimating the actual effect of marriage and of different types of marriage and would make us accept wrong hypotheses. In the next section we present our identification strategy.

## METHOD

We handled the selection issues using propensity score matching and a data set rich on covariates. Propensity score matching efficiently accounts for covariates that affect selection into marriage and different types of marriage by balancing the samples of treated participants and controls on all relevant observed variables (Rosenbaum & Rubin, 1983). In addition, it balances the samples on unobserved variables that are correlated with the observed variables to the extent that this correlation is the same in the two samples. By assumption, this conditioning on all relevant individual characteristics leaves treatment allocation—whether a person marries or not—random (this is the *conditional independence assumption*), which allows us to claim that the effect of marriage is unbiased.

To reduce the risk of bias originating from unobserved and confounding characteristics, we balanced our samples on a range of covariates that far exceeds what is typically seen in the literature. Our focus on recidivism rather than crime in general also means that we included only previously convicted men in the analysis. This prevented bias from unobserved differences between convicted and never‐convicted men from influencing our results, just as it rid our sample of men who are not very likely to commit future crimes and who then function only as statistical noise (Kurlychek, Brame, & Bushway, 2006). Second, we included measures of individual outcomes before treatment (prior criminality) in our propensity score model. This strategy served two functions by adjusting for the observed criminal history of the convicted men in our analysis and for unobserved factors correlated with this criminal history that might otherwise have affected selection into marriage and into different marriages (Dehejia & Wahba, 1999, 2002; Heckman, Ichimura, Smith, & Todd, 1998; Heckman, Ichimura, & Todd, 1997).

We estimated the propensity score using a standard logit estimator, and we restricted our analysis to observations that have common support, as is customary. We present results from 1:1 nearest neighbor matching with replacement. With this strategy we matched each married man in our sample to the unmarried man with the most identical propensity score, and we allowed each unmarried man to function as control observation for several married men.

### *Data*

We analyzed the effect of marriage on recidivism using administrative data from Statistics Denmark. In Denmark, all residents have a unique personal number that identifies the residents in a great many transactions, such as interaction with the welfare system, schooling, and work status. Statistics Denmark makes these data available for statistical and research purposes, and the panel goes as far back as 1980. These data provided information on who is married to whom, which means that we could link spouses; we even knew the exact date of the marriage. In addition, the data also contained information on all criminal justice contacts, and they were therefore highly suited for testing the effect of marriage on criminal involvement.

From the data, we selected a sample of all men born between January 1965 and December 1985 (730,986 men). These were the cohorts who turned 18 (the legal marriage age in Denmark) during our data period, which spanned from 1980 until 2005, and whom we could follow from the beginning of their criminal career (the age of criminal responsibility in Denmark is 15). The later cohorts were obviously followed for fewer years, but they still meet the criteria of being able to marry before the end of our data period in 2005. To get a clean assessment of the marriage effect and because we analyzed only the effect of first marriages, we excluded men who had already been married or were married in December of 2000 (this reduced our sample to 539,680 men), and we excluded observations due to migration and death. This reduced our sample to 480,968 men. Of these, we kept only men who had been convicted at least once prior to 2000 and those whom we could follow in the registers for at least 3 years prior to their marriage date (103,412 men). From this sample we deleted eight men sentenced to undergo psychiatric treatment and 565 men for whom we had incomplete information on their future spouse. Our final analytic sample then consisted of 102,839 previously convicted and never‐married men as of 2000.

Our sample had four key advantages compared to the samples used by most studies on marriage and crime. First, the individual‐level data allowed us to assess the individual‐level relationship between marriage and recidivism rather than aggregate correlations. Second, the population data removed problems related to attrition. Third, the several available and linkable registers allowed us to add a wide range of covariates to our sample, which was required for fulfilling the conditional independence assumption in propensity score matching that all selection occurred on observed variables. Fourth and finally, the official nature of our data implied that we had precise information on all convictions and dates of offenses rather than less precise self‐reports often found in surveys (for a discussion of official data vs. self‐reports, see Kirk, 2006).

### *Variables*

#### Treatment

To investigate our two hypotheses, we needed to specify three different treatments, (a) marriage (Treatment 1), (b) marriage to a nonconvicted spouse (Treatment 2), and (c) marriage to a convicted spouse (Treatment 3). Individuals who received Treatments 2 or 3 always also received Treatment 1, but there was no overlap between Treatments 2 and 3. The first treatment concerned all men in our sample who married for the first time between 1/1/2001 and 12/31/2004. We used this relatively large time frame for marriage dates to ensure that we had enough observations in the different types of treatment (see below) for the distributional assumptions to hold. Nine thousand, five hundred seventy‐five men in our sample received this treatment. The second treatment concerned the subgroup of these 9,575 men who married a nonconvicted woman, specified as a woman who had not been convicted during the 5 years preceding the marriage (note, however, that our results were robust to different specification of this time frame). Nine thousand, two hundred sixty‐six men received this treatment. Our last treatment concerned the subgroup of Treatment 1 who married a spouse who had been convicted at some point during the 5 years preceding the marriage. Only 309 men received this treatment. Given the theory on assortative mating, this number seems low, but it reflects that only a few Danish women are convicted of crimes and that criminal past is not the only dimension along which people mate.

We considered the effect of these three treatments in four different models. First, we assessed the effect of each of the three treatments against a control group of the same previously convicted 93,264 men who did not experience their first marriage before 12/31/2005. These men had not married prior to our observation period, and they did not marry during it, and the counterfactual outcome for all treatments in these three models was thus staying unmarried. We ran these three models to test Hypothesis 1 (Treatment 1) and to understand whether marriage in the two forms (marriage to a convicted spouse and marriage to a nonconvicted spouse) made a difference in terms of desistance when not marrying represented the counterfactual outcome. Second, we tested Treatments 2 and 3 against each other, and in this model the two treatments were considered each other's counterfactual. We ran this last model to test the relative difference between our two key treatments (which was implied by Hypothesis 2). This showed whether it was better for previously convicted men to marry a nonconvicted woman rather than a woman with a criminal history.

#### Dependent variable

Our dependent variable was a binary indicator of *criminal recidivism*, measured as whether or not a person had committed new crime during a 12‐month follow‐up period, and we considered only offenses that eventually led to conviction. To avoid the risk that queues at court could skew our results, only the offense needed to occur during the first 12 months following the marriage date; the conviction did not. For the treatment groups (those who married), the outcome variable measured criminal recidivism during the 12 months following the marriage date (between 1/1/2001 and 12/31/2004). The men in our control group did not have an actual treatment date (because they did not marry), and we therefore assigned these males the pseudotreatment date of 1/1/2003 and observed their criminal activity during the subsequent 12 months (until 1/1/2004). For all groups, the outcome variable then indicated any criminal offense recorded during an entire year, which removed possible problems of seasonal variation in crime patterns.

Note that we observed the 1‐year recidivism of the control group for the calendar year (2003) that lay in the middle of the period in which we observed the 1‐year recidivism of each person in the treatment group (2001–2005). Hence, variations in the business cycle during the years 2001–2005 may cause differences in the 1‐year recidivism rates of the treatment and the control groups. We tested to see whether our results were sensitive to changing the pseudotreatment date of the control group and found that this was not the case.

#### Control variables

We examined three types of treatment: (a) marriage in general, (b) marriage to a nonconvicted spouse, and (c) marriage to a previously convicted spouse. Our choice of covariates addressed the selection into each of these three marriage types.

First, we included two measures of family formation events other than marriage that might occur in the years prior to the treatment, namely, cohabitation and childbirth. We measured *cohabitation* as the number of years the man had cohabited with any woman and *childbirth* as whether the man had children or not (not necessarily with his future wife). Both events are strong predictors of the propensity to marry because they show commitment to social norms. In addition, they signal marriage potential by indicating that the man is experienced with such institutions. Although cohabitation and parenthood are important controls in the propensity score for the probability of marriage, it is less clear how they affect the selection into types of marriages. One might suggest that the signaling value of engaging in these social institutions could increase a man's likelihood of marrying a nonconvicted spouse, given that it reveals the ability to conform to society's norms.

Second, we included *age* and *age squared*. Age is an important control because it influences both marital transitions and criminal activities (e.g., Hirschi & Gottfredson, 1983), and the level of maturity signaled through a man's age may also increase his probability of marrying a law‐abiding spouse.

Third, we included a range of variables on socioeconomic characteristics: labor market affiliation, income, educational attainment, ethnicity, geographic location, and an indicator of whether the man comes from a single‐parent household. We measured *labor market affiliation* as the mean unemployment rate during the previous 3 years, *income* as mean annual income during the previous 3 years—including the squared mean income to give more weight to income in the propensity score—and *educational attainment* as years of education. Regarding *ethnicity*, we distinguished among Western immigrants, non‐Western immigrants, and people of Danish origin. We measured *geographic location* as the population density in the municipality of residence. Last, we indicated *whether the man grew up in a single‐parent household* by measuring the parents' marital status when he was 15. These variables on socioeconomic characteristics are important predictors of marriage, as the literature emphasizes how personal resources increases the man's marriage potential (e.g., G. Becker, 1991). In addition, the acquisition of personal resources is also part of the same process that prepares the individual for marriage. Having a stable labor market affiliation and a steady income may signal readiness for stability in other areas, such as marriage (Oppenheimer, 1988). Moreover, these characteristics also matter for the individual's motives and incentives for engaging in criminal activities (Rhule‐Louie & McMahon, 2007). The different socioeconomic characteristics may then also influence the probability of marrying a nonconvicted spouse rather than a convicted one due to assortative mating, as already discussed.

Fourth, we included information on criminal history. Although all men in our sample had previously been convicted, we further controlled for the extent of their previous crimes. We included *age at criminal onset*, an indicator of *prior imprisonment*, and the *number of convictions* within each of the 3 years prior to the marriage. This information on the man's most recent crimes might be a signal of marriage potential (King, Massoglia, & MacMillan, 2007). Also, as argued above, the theory of assortative mating predicts that a man's criminal past will affect his propensity to marry a nonconvicted versus a convicted spouse.

Fifth, we included information on the *number of previous convictions of violating the law on illicit drugs*. Such charges reflect the possession of small amounts of drugs, not for resale or distribution, and are useful proxies for drug abuse. According to the literature, drug abuse correlates with important unobserved characteristics, such as sensation seeking, inconsistency, and other attributes that may or may not be considered desirable by the different women (Rhule‐Louie & McMahon, 2007). Hence, drug abuse will affect selection into marriage, and it might lower one's propensity to marry a nonconvicted spouse.

Descriptive statistics of the included covariates, by marital status, are shown in Table 1. Notice how the married and unmarried men in our sample differed on all characteristics except from the share who came from single‐parent households. Also, as expected, those who married were older, more educated, and had a higher income and lower unemployment. They lived in less populated areas and had longer cohabitation spells, and a larger share had kids. In terms of criminal history, those who did not marry were generally more active than those who married.

**Table 1:** Descriptive Statistics of Covariates, by Marriage Status

Variable	Not married (*n =* 93,264)		Married (*n =* 9,575)	
	*M*	*SD*	*M*	*SD*
Age	27.70	5.68	29.50	4.03***
Years of education	11.37	2.71	12.20	2.61***
Missing information on education	.02	.13	.01	.10***
Income (in DKK 1,000)^a^	169.21	109.48	237.00	135.17***
Unemployment rate	.08	.15	.05	.12***
Local population density > 700	.29	.45	.27	.44***
Local population density 150–700	.37	.48	.35	.48***
Western origin	.01	.08	.00	.06**
Non‐Western origin	.03	.16	.02	.13***
Years of cohabitation	2.17	3.13	4.43	3.42***
Has kids	.22	.42	.37	.48***
Age at criminal debut	19.02	3.71	19.85	4.11***
Committed crime, last year	.21	.54	.08	.32***
Committed crime, 2 years ago	.20	.53	.07	.31***
Committed crime, 3 years ago	.20	.53	.06	.29***
Previous convictions for drugs	0.41	1.14	0.20	0.72***
Ever imprisoned	.55	.50	.42	.49***
Parents married at age 15	.82	.39	.82	.39
Parents' marital status missing	.00	.07	.00	.06

a At the time of this writing, 1,000 DKK ≈ 167 USD.

** *p* < .01.

*** *p* < .001.

## RESULTS

The results from our four propensity score models, which estimated the probabilities that the men in our sample experienced either one of the treatments, are presented in Table 2. Model 1 shows the general marriage propensity, Model 2 shows the propensity for marrying a nonconvicted spouse, and Model 3 shows the propensity for marrying a previously convicted spouse. The lower number of observations in Model 3 reflects that this model excluded men receiving Treatment 2. The control group was the same in these three models and consisted of men who did not marry in our observation period, and the counterfactual outcome was staying unmarried. In Model 4 we tested Treatments 2 and 3 against each other. Here, men who received Treatment 2 acted as controls for men who received Treatment 3, which means that we compared the recidivism rate of men who married a previously convicted spouse to the recidivism rate of men who married nonconvicted spouses.

**Table 2:** Logit Coefficients of the Propensity Score Models

Variable	Model 1 (*n =* 102,839)		Model 2 (*n =* 102,530)		Model 3 (*n =* 92,982)		Model 4 (*n =* 9,540)	
	*B*	*SE*	*B*	*SE*	*B*	*SE*	*B*	*SE*
Age	1.20	0.03***	1.20	0.03***	0.80	0.13***	−0.35	0.15**
Age squared	−0.02	0.00***	−0.02	0.00***	−0.01	0.00***	0.01	0.00**
Years of education	0.07	0.01***	0.07	0.01***	−0.11	0.03***	−0.16	0.03***
Missing educational information	0.78	0.13***	0.74	0.14***	−0.53	0.42	−1.29	0.50***
Income	0.00	0.00***	0.00	0.00***	−0.00	0.00***	−0.01	0.00***
Income squared	−0.00	0.00***	−0.00	0.00***	0.00	0.00**	0.00	0.00***
Unemployment rate	−1.13	0.10***	−1.16	0.10***	0.09	0.34	0.61	0.40
Local population density > 700	−0.15	0.03***	−0.14	0.03***	−0.49	0.16***	−0.22	0.18
Local population density 150–700	−0.09	0.03***	−0.09	0.03***	−0.21	0.13	−0.04	0.14
Western origin	−0.31	0.18*	−0.27	0.18				
Non‐Western origin	0.50	0.08***	0.50	0.09***	0.50	0.30*	−0.29	0.35
Years of cohabitation	0.15	0.00***	0.15	0.00***	0.08	0.02***	−0.07	0.03**
Has kids	0.08	0.03***	0.08	0.03**	0.45	0.15***	0.19	0.15
Age at criminal debut	0.03	0.00***	0.04	0.00***	0.04	0.02**	0.01	0.02
Committed crime, last year	−0.24	0.04***	−0.37	0.04***	0.21	0.08***	0.41	0.11***
Committed crime, 2 years ago	−0.29	0.04***	−0.48	0.05***	0.29	0.07***	0.58	0.12***
Committed crime, 3 years ago	−0.39	0.04***	−0.58	0.05***	0.20	0.08***	0.67	0.12***
Previous convictions for drugs	−0.08	0.02***	−0.11	0.02***	0.05	0.03*	0.17	0.05***
Ever imprisoned	−0.20	0.02***	−0.21	0.02***	0.27	0.14*	0.42	0.15***
Parents married at age 15	0.08	0.03***	0.08	0.03***	−0.11	0.14	−0.12	0.15
Parents' marital status missing	−0.11	0.18	−0.11	0.19	0.07	0.73	0.59	0.77
Model intercept	−20.43	0.48***	−20.56	0.50***	−15.96	1.84***	3.72	2.13*
Pseudo *R* ^2^	.13		.14		.05		.21	
Log likelihood	−27,806.81		−26,828.76		−1,959.92		−1,082.13	
χ^2^	8,078.12		8,557.93		223.95		563.31	

* *p* < .05.

** *p* < .01.

*** *p* < .001.

Because of the large number of coefficients, we do not describe them in detail, but readers should notice how all estimates point in the expected directions. For example, being in the labor market was important for marrying a nonconvicted woman, whereas it mattered only little for the propensity to marry one who had previously been convicted. Also, although previous crime did not seem to matter for a man's probability of marrying a previously convicted woman, it was negatively correlated with marrying a nonconvicted woman. Last, we see that men who lived in scarcely populated areas were more likely to marry convicted women.

### *Matching Results*

The first row of Table 3 shows our matching results. Models 1, 2, and 3 show the estimated effects of each of the three treatments relative to staying unmarried, and Model 4 shows the estimated effect of marrying a convicted spouse compared to marrying a nonconvicted spouse. These models provided us with (a) the overall estimated effect of marriage relative to nonmarriage (Model 1); (b) the estimated desistance effect of the two types of marriage relative to nonmarriage (Models 2 and 3); and (c) differences in the estimated desistance effect of these two types of marriage, which showed whether marriage to a nonconvicted spouse was more likely to facilitate desistance than marriage to a convicted spouse (Model 4). The samples were perfectly balanced on all covariates in all models (see Appendix Table A1).

**Table 3:** Matching Results on the Effect of Marriage on Recidivism, by Spousal Criminality

Model description		Model 1^a^	Model 2^b^	Model 3^c^	Model 4^d^
	Main analysis				
Difference in recidivism, matched samples	Estimate	−.02***	−.02***	.03	.11***
	*N* (all / treated)	93,264 / 9,574^e^	93,264 / 9,265^f^	93,264 / 309	9,231^g^ / 309
Difference in recidivism,unmatched samples	Estimate	−.08***	−.09***	.13***	.22***
	*N* (all / treated)	93,264 / 9,575	93,264 / 9,266	93,264 / 309	9,266 / 309
	Sensitivity analysis				
Spousal criminality observed 0–2 years prior to marriage	Estimate	−.02***	−.02***	.06	.08*
	*N* (all / treated)	93,264 / 9,574	93,264 / 9,419	93,264 / 156	9,384 / 156
Spousal criminality observed 0–8 years prior to marriage	Estimate	−.02***	−.02***	.02	.06***
	*N* (all / treated)	93,264 / 9,574	93,264 / 9,073	93,264 / 502	9,073 / 502

a In Model 1 the treatment is marriage (to any spouse) and the control is nonmarriage.

b In Model 2 the treatment is marriage to a nonconvicted spouse and the control is nonmarriage.

c In Model 3 the treatment is marriage to a convicted spouse and the control is nonmarriage.

d In Model 4 the treatment is marriage to a convicted spouse and the control is marriage to a nonconvicted spouse.

e One observation is off common support and thus not included in the analysis.

f One observation is off common support and thus not included in the analysis.

g Thirty‐five observations are off common support and thus not included in the analysis.

* *p* < .05.

*** *p* < .001.

From Model 1 we learned that marriage reduced recidivism. On average, an estimated 2 percentage points fewer of the previously convicted men recidivated during the year following their marriage date than comparable men who remained unmarried. This finding corresponded to the findings of previous studies of the marriage effect, and it reinforced the predictions of theories on marriage and desistance, which state that marriage promotes desistance by facilitating social control and reducing interaction with delinquent peers.

Our estimate of the protective effect of marriage may seem small, given that the recidivism rate is only 2 percentage points lower during the first year following marriage. Still, with a strong correlation between previous and future convictions (Kurlychek et al., 2006), even a small estimated effect influenced the aggregate crime level in our sample, in which all men were previously convicted. In fact, our estimate suggested that marriage prevented as many as 163 men in our treatment group from recidivating (9,575 × 0.02 = 162.8, calculated from the unrounded parameter estimate) and could have prevented 1,585 recidivists in the control group, had they married (assuming that treated and controls respond similarly to marriage).

From Model 2 we learned that marriage to a nonconvicted woman also significantly reduced crime. According to our estimates, the share of recidivists among previously convicted men who married nonconvicted women was also 2 percentage points lower than among the control group of comparable men who did not marry. This comes as a small surprise given that this type of marriage made up the largest share of the marriages in our sample. The allocation of this treatment to 9,266 previously convicted men thus reduced the aggregate number of criminal recidivists by 185 persons.

Model 3 shows the estimated effect of our third treatment specification: marrying a convicted woman. This estimate was positive and numerically larger than the estimated protective effect of marriage to a nonconvicted spouse. It is important to note, though, that the estimate was not statistically significant. This lack of statistical significance could arise from the small number of men who got this treatment and may not correspond to substantial insignificance, but these are mere speculations. Our results hence provided no evidence that marriage to a previously convicted spouse increased or decreased recidivism levels for this group of men compared to previously convicted men who did not marry.

It is interesting that the result from Model 4 was both positive and significant. This shows that, compared to the men in our sample who married a nonconvicted spouse, men who married a convicted spouse were more likely to recidivate during the follow‐up period. The estimated difference was a nonnegligible 11 percentage points. This corresponds to 34 additional recidivists among men marrying a convicted spouse compared to men who married nonconvicted spouses. This result followed our theoretical predictions and confirmed Hypothesis 2 by suggesting that it is better for previously convicted men to marry a nonconvicted woman than to marry a previously convicted woman.

The second row of Table 3 shows the unbalanced differences in recidivism rates between treated and control men, calculated as the simple bivariate correlation between marriage type and crime. These were the non‐bias‐corrected mean differences that reflected both the estimated marriage effect and the selection of different men into marriage and into different types of marriage. We present these results to illustrate the implications of not accounting for selection, and, as expected, the “marriage effects” here were numerically much larger simply because they also reflected bias arising from the selection of specific men into specific types of marriages. These results are not useful for understanding the marriage effect but instead serve to highlight why it is important to account for selection.

### *Sensitivity Analysis*

In our main results we defined *spousal criminality* as the spouse having been convicted within the previous 5 years. The choice of timeframe in the definition of spousal criminality was arbitrary, and we tested whether this choice mattered for our results. The two bottom rows of Table 3 show results from this sensitivity analysis in which we used different time windows for assessing the wife's criminality. The first row shows results when we defined spousal criminality as crime committed 0 to 2 years prior to the marriage, and the second row shows results for spousal criminality committed 0 to 8 years prior to the marriage. As can be seen, our main results were robust to changes in the time window.

One concern that often accompanies the use of propensity score matching is whether results are sensitive to the applied matching algorithm. Our main results relied on 1:1 nearest neighbor matching, which is the most straightforward algorithm. But our results were robust to the choice of matching algorithm, as the findings of both 1:10 nearest neighbor matching and kernel matching—which replicates the estimation strategy in King et al. (2007)—supported our conclusions (results available on request).

As discussed, another important concern that always accompanies estimates based on propensity score matching is whether the model appropriately accounted for selection on unobservables, that is, whether we fulfilled the conditional independence assumption. We addressed this concern by calculating Rosenbaum bounds (results not shown but available on request).

Rosenbaum bounds are calculated by introducing bias of increasing magnitude into our main model. The idea was to assess how strongly an unobserved variable must affect the selection process in order to undermine our conclusions. Thus, Rosenbaum bounds allowed us to assess the strength of our results against unobserved bias by showing the minimum bias required for turning these results statistically insignificant. In Model 1 and Model 2 we suspected negative (unobserved) selection, because men who were most likely to marry and to marry nonconvicted women were also those who were most likely to desist, such that we might have overestimated the true marriage effect. From our Rosenbaum bounds calculations we learned that such bias needs to exceed more than double the odds of marriage in order to alter our conclusion regarding the general marriage effect. Likewise, our results in Models 3 and 4 required bias of more than three times the odds of marriage to alter our conclusions.

The Rosenbaum bounds thus uniformly showed that our models were very robust against unobserved variables. Also, even though it is important to note that Rosenbaum bounds do not offer a direct test of the conditional indepen‐dence assumption (see S. Becker & Caliendo, 2007), our calculations did revoke the suspicion that our results could be driven by unobserved selection.

## DISCUSSION

In this study we used unique administrative data and propensity score models to estimate the effect of marriage on criminal recidivism and to estimate whether this effect depends on the criminal profile of the spouse. Overall, we found that marriage reduced recidivism, echoing existing studies on the marriage effect. But the protective effect of marriage on recidivism also depended on the criminal profile of the wife. Our results showed that convicted men who married convicted women were significantly more likely to recidivate than convicted men married to nonconvicted women. Our study is the first to demonstrate such heterogeneous marriage effects on recidivism, and even though it may have been expected given existing empirical studies as well as theories on marriage effects, the study adds to the literature by applying a rigorous methodological approach and using very rich data.

Our results have two interesting implications. First, they show that one cannot just rely on social institutions to decrease recidivism because they often represent heterogeneous types of treatment. It is not the marriage but rather the type of marriage that matters. Second, our results show that we get empirically valid hypotheses from an extrapolation of existing theories on the marriage effect into the analysis of the effect of marriage to women with different criminal histories. This enforces the explanatory power and thus the empirical validity of the theories.

Marriage in most Western countries is currently undergoing substantial changes and has been changing fundamentally since the 1950s. Now, marriage rates are at their lowest since 1950, and the median age at first marriage has risen dramatically. These developments may impair the use of our results. It is important to note, though, that we used Danish data, and Denmark is typically seen as one of the forerunners in the Second Demographic Transition (Sobotka, 2008), and although Denmark differs from most other developed countries on a range of parameters, our findings could still reflect the future of these other countries in terms of marriages. Our results on how the marriage effect on recidivism varies by spousal criminality in contemporary Denmark might in this way serve as a projection of how this effect will unfold in the future in other developed democracies.

Furthermore, the penal system in Denmark is often considered quite lenient, particularly when compared to the United States. The United States has the world's highest imprisonment rate (743 per 100,000 inhabitants in 2011), whereas Denmark is among the countries with the lowest (74 per 100,000 inhabitants in 2011; Walmsley, 2011). In addition, as many as 85% of sentences in Denmark are shorter than 1 year, and less than 7% are longer than 2 years (2011 levels; Danish Prison and Probation Service, 2012). In contrast, the mean sentence length in the United States is around 4.7 years for federal prisoners (2008–2009 level) and around 2.1 years for state prisoners (Guerino, Harrison, & Sabol, 2011; Motivans, 2011). The social impact of being convicted and sentenced in those two contexts will thus most likely differ. But, because we have shown that marriage had an effect on recidivism in a lenient country such as Denmark, this effect is all the more likely to exist in other contexts, such as in the United States, where social divides and penal consequences are much stronger.

With our results, we still do not know whether it is the lack of informal social control or the maintenance of a network of criminal peers that explains failed desistance among men married to convicted women. Future research should therefore try to separate out the mechanisms of the two theories. And even though we took a long line of steps to minimize the risk of selection issues in our results—by balancing our samples using a wide range of control variables, including pretreatment outcome variables that accounted for unobserved heterogeneity in our propensity score model and by looking only at previously convicted men—whether one is willing to place trust in results from propensity score matching is fundamentally a matter of opinion. Future studies should search for valid instruments that effectively randomize marriages, because this is the only feasible way to obtain causal estimates that are less contaminated than ours might be.

## NOTE

We thank the Rockwool Foundation Research Unit for funding support. This article has benefited greatly from the comments and critiques of Jukka Savolainen, Mads Meier Jæger, Richard Breen, Steven Messner, Torbjørn Skardhamar, Susumu Imai, Søren Leth‐Petersen, Elizabeth Roberto, Annette Fasang, Nicholas Hoover, Anders Holm, Kristian Karlsson, Vibeke Tornhøj Christensen, and Claus Larsen. Any mistakes remain our own.

## Appendix

**Table A1:** Statistics (t‐Tests) of Covariates in the Matched Samples, by Treatment Variable

Variable	Model 1 (*n* = 102,839)	Model 2 (*n* = 102,530)	Model 3 (*n* = 92,982)	Model 4 (*n* = 9,540)
Age	−0.73	−0.73	0.16	0.52
Age squared	−0.75	−0.73	0.15	0.46
Years of education	0.18	0.02	−0.16	0.56
Missing educational information	−0.06	−0.11	0.23	−0.31
Income	−0.02	−0.00	0.35	−0.02
Income squared	−1.18	−1.39	1.18	0.48
Unemployment rate	1.11	0.50	−0.43	−0.65
Local population density > 700	0.06	0.34	−0.10	0.23
Local population density 150–700	0.26	−0.27	−0.05	0.04
Western origin	−0.12	0.12		
Non‐Western origin	−0.42	−0.56	−0.22	−0.78
Years of cohabitation	−0.82	−0.64	−0.11	0.47
Has kids	−0.42	−0.40	0.02	0.14
Age at criminal debut	0.41	0.46	−0.04	−0.18
Committed crime, last year	−0.19	−0.19	0.50	0.24
Committed crime, 2 years ago	−0.33	−0.36	−0.05	0.71
Committed crime, 3 years ago	−0.87	−0.10	−0.18	0.90
Previous convictions for drugs	0.11	0.07	0.04	0.26
Ever imprisoned	−0.48	−0.37	0.34	−0.30
Parents' married at age 15	0.06	0.13	0.50	−0.23
Missing parents' marital status	0.14	0.27	−0.00	−0.24
